# Editorial: Exercise intervention in cancer management and rehabilitation: a spotlight on exercise oncology

**DOI:** 10.3389/fphys.2023.1215763

**Published:** 2023-06-06

**Authors:** Junga Lee

**Affiliations:** Graduate School of Sport Science, Global Campus, Kyung Hee University, Seoul, Republic of Korea

**Keywords:** cancer, management, rehabilitation, technology, exercise

## Introduction

Exercise is important for cancer survivors. Several previous meta-analysis studies regarding the effectiveness of exercise interventions reported improved quality of life and pro-inflammatory markers in breast and prostate cancer survivors (Bates-Fraser et al., 2023; Khosravi, Stoner, Farajivafa, & Hanson, 2019). These studies also reported a reduction in cancer-related fatigue in colorectal cancer survivors, and enhanced muscle strength, physical activity, body mass index, and fatigue in childhood cancer survivors (Machado et al., 2022; Shi, Zheng, & Liu, 2022). Understanding the effects of exercise interventions helps illustrate why individuals need to participate in exercise and provides health-related professionals, including oncologists and exercise specialists, with insight into cancer mortality, cancer recurrence, and treatment effects for cancer survivors. To increase the effects of exercise interventions through technologically advanced approaches, there is a clear need to explore access to technology and how these might be applied to cancer survivors. We investigated the additional effects of exercise interventions focusing on health-related quality of life, physical fitness, and body composition in breast cancer patients. To increase the effects of exercise interventions as a cancer treatment in a clinical oncology setting, a guideline of exercise interventions is presented for clinical oncology as a practical and simple taxonomy. Finally, a survey study on technology access and preferences regarding exercise interventions was performed.

### Effects of exercise interventions

The effects of exercise interventions for cancer survivors have been shown to convey beneficial effects. Several studies have reported evidence of the effects of exercise, including health-related factors, such as immunity, quality of life, physical fitness, and body composition in diverse cancers including prostate cancer, breast cancer, and colorectal cancer (Ferrer, Huedo-Medina, Johnson, Ryan, & Pescatello, 2011). Depending on the cancer type, the progression and side effects experienced by cancer survivors were diverse. This Research Topic provides additional insights from recent studies, outlining the favorable effects of exercise interventions that are considered the typical signs of breast cancer survival, such as decreased cardiovascular fitness and increased fatigue compared with other cancers. Joaquim et al. conducted a systematic review and meta-analysis of the effect of physical exercise on health-related quality of life, cardiorespiratory fitness, muscle strength, and the body composition of breast cancer survivors. They observed a moderate, short-lived effect and heterogeneity among studies. The suggestions for future study in this review and meta-analysis were to investigate the effects of exercise interventions for breast cancer survival depending on the exercise type, frequency, timing, and adverse events. Additional studies with detailed descriptions of exercise interventions, cancer type, and cancer treatment among participants will help to establish such interventions for cancer treatment.

### Exercise guidelines for cancer treatment

To date, there have been diverse suggestions for exercise guidelines relating to potential cancer treatments. Having exercise guidelines based on evidence-based studies would be beneficial and practical in a clinical oncology setting. In this Research Topic, Courneya; Friedemreich have presented exercise as a cancer treatment: a clinical oncology framework for exercise oncology research. The purpose of this study was to suggest a conceptual framework for exercise guidelines in the setting of distinct clinical oncology and to review overall exercise studies using the Exercise as Cancer Treatment (EXACT) framework that was proposed for nine distinct clinical oncology scenarios, thereby tailoring exercise based on the status of tumor/disease and status of treatment. This study suggested a simple taxonomy, the EXACT framework, which recommends treatment protocols for different cancer types. Studies of exercise interventions as cancer treatments using the proposed framework covered the most common clinical cases. Further study on a larger number and greater diversity of patients is needed.

### Future exercise interventions

Home-based exercise interventions are optional for cancer survivors. Increased technology helps to enable remote exercise interventions at any time and place when cancer survivors want to participate. Diverse technology is applied for home-based exercise interventions. However, there is an increasing disparity in technology access between urban and rural survivors, possibly resulting in skewed survival between such residents. Salerno et al. conducted a survey to understand access to technology and the preferences of cancer survivors in rural locations. This survey reported that most rural residents preferred interventions delivered by computer or mobile phone that included balance, flexibility, and aerobic exercise two or three times a week for at least 20 min. Rural cancer survivors also preferred a tailored exercise regimen that considered their health status. A further study will be conducted with a larger sample size and different methodologies to increase the reliability of this survey.

## Conclusion

This Research Topic summarizes exercise interventions in cancer management and rehabilitation based on previous evidence-based studies and proposes a clinical oncology framework for exercise interventions. Additionally, to develop further effective exercise interventions, rural cancer survivors’ access to technology and preferences should be considered. These and similar studies provide a foundation to improve the experience of cancer survivors who participate in exercise interventions as part of cancer management and rehabilitation.

